# The relationship between dietary inflammatory index in adults and coronary heart disease: from NHANES 1999–2018

**DOI:** 10.3389/fnut.2025.1564580

**Published:** 2025-03-21

**Authors:** Hong Xu, Pengxin Xie, Hui Liu, Zhenyu Tian, Ruitao Zhang, Ming Cui

**Affiliations:** ^1^College of Science, Minzu University of China, Beijing, China; ^2^Department of Cardiology and Institute of Vascular Medicine, Peking University Third Hospital, Beijing, China; ^3^State Key Laboratory of Vascular Homeostasis and Remodeling, Peking University Third Hospital, Beijing, China

**Keywords:** dietary inflammatory index, coronary heart disease, National Health and Nutrition Examination Survey, mediating factors, interaction

## Abstract

**Background:**

Previous studies have shown that pro-inflammatory diets increase the risk of coronary heart disease (CHD) and all-cause mortality. The dietary inflammatory index (DII) is a quantitative measure of dietary inflammation, and its accuracy has been validated by several studies.

**Methods:**

This study included 43,842 participants aged ≥18 years from the National Health and Nutrition Examination Survey (NHANES) 1999–2018. The data of CHD was obtained through a questionnaire survey, and the DII was calculated using 24-h dietary recall data. Generalized linear models and logistic regression were used to determine the mediation factors, and subgroup analyses were conducted to evaluate the interaction between DII and CHD. Mean decrease in Gini (MDG) was used to determine the importance of individual dietary components.

**Results:**

The age of the participants was 49.81 ± 18.10 years, with 20,793 (47.4%) being male. A total of 1,892 (4.3%) participants were diagnosed with CHD, and the median DII score was 1.33 (0.11, 2.40). After adjusting for potential confounders, logistic regression analysis revealed that DII independently associated with CHD [OR: 1.049 (1.012–1.087), *p* = 0.008]. Triglyceride-glucose index, visceral adiposity index, body mass index, waist-to-height ratio, high-density lipoprotein, and glomerular filtration rate (all *p* < 0.05) may mediate the relationship between DII and CHD. Subgroup analyses showed that DII was more sensitive in participants aged <75 years (*p* < 0.001), females (*p* = 0.028), those with low cholesterol levels (*p* = 0.004), and individuals with low Framingham risk scores (*p* = 0.005). MDG analysis indicated that carbohydrate, vitamin C and iron intake have the greatest impact on CHD.

**Conclusion:**

This study suggests that various metabolic and lipid indicators play a mediating role in the relationship between DII and CHD. DII may have a greater adverse impact on traditional low-risk CHD populations.

## 1 Introduction

Coronary heart disease (CHD) is characterized by accumulation of lipids and fibrous tissue in the intima of the arterial wall, smooth muscle cell proliferation, and local and systemic inflammatory responses (1). CHD poses a giant threat to health. In the United States, approximately 20.1 million individuals are diagnosed with CHD (2). Despite recent advances in treatment, it remains one of the leading causes of death globally (3). The complex pathophysiological mechanisms of CHD present a significant challenge to improving patient outcomes. Studies have shown that inflammation plays a key role in the onset and progression of CHD (4). It is involved in several stages of atherosclerosis initiation and progression, plaque rupture, ischemia–reperfusion injury, and ventricular remodeling (4).

Healthy dietary habits, such as the Mediterranean diet, high-fiber diets, and plant-based diets, have been shown to be associated with lower systemic inflammation levels. The dietary inflammatory index (DII), first proposed by Cavicchia et al. (5), is used to quantitatively assess the inflammatory potential of diet. The DII is based on 45 different anti-inflammatory and pro-inflammatory food components. A positive DII score indicates a pro-inflammatory effect, while a negative score indicates an anti-inflammatory effect. The higher the score, the more pronounced the pro-inflammatory effect. An increasing body of evidence has described a certain correlation between higher DII and elevated levels of inflammatory markers, such as hypersensitive C-reactive protein (hsCRP), tumor necrosis factor-*α* (TNF-α) and interleukin-6 (IL-6) (6–8).

Current research suggests that the DII is associated with subclinical atherosclerosis. DII in patients with CHD was significantly higher than in those without CHD (9). A 36-month follow-up study found a positive association between DII and the risk of atherosclerosis-related mortality [HR: 1.36(1.15–1.60)] (10). Additionally, DII was positively associated with all-cause mortality risk in CHD patients, particularly among women (11). However, current studies have not explored the interaction between DII and traditional risk factors, nor have they investigated potential mediating factors between DII and CHD. Due to the inclusion of multiple variables, guiding patients on diet based on DII becomes more complex.

Therefore, this study aims to explore the correlation between DII and CHD, as well as traditional risk factors, further analyze mediating factors and interactions, and rank the importance of dietary components to enhance the practicality in clinical settings.

## 2 Methods

### 2.1 Study design and population

**Figure 1:**
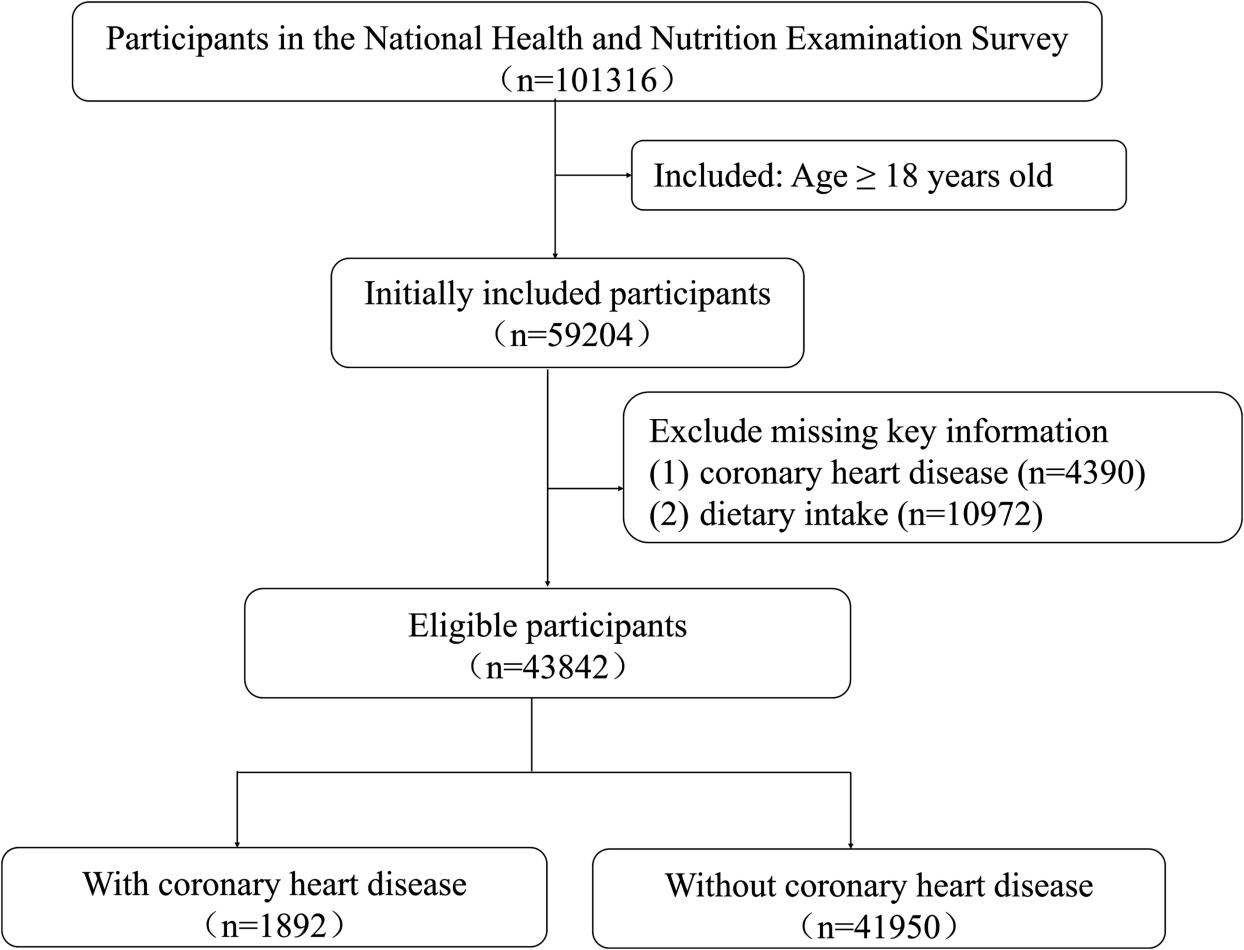
Flowchart of the study population.

The National Health and Nutrition Examination Survey (NHANES) is a research project aimed at assessing the health and nutritional status of United States. The survey used scientific sampling methods and annually collected data from approximately 5,000 individuals from 15 counties, representing the national population. The inclusion criteria for this study were: (1) participants in NHANES surveys from 1999 to 2018; (2) age ≥ 18 years. The exclusion criteria were: (1) missing key variables regarding the presence of CHD; (2) missing dietary data required for the calculation of the DII. Ultimately, 43,842 participants were included in this study. The detailed participant inclusion process was shown in Figure 1. The NHANES study was approved by the Institutional Review Board of the National Center for Health Statistics, and all participants provided written informed consent.

### 2.2 Dietary inflammatory index (DII)

The DII was calculated based on individual dietary components, with dietary data collected through 24-h dietary recall interviews in NHANES. For the 1999–2002, only 1 day’s dietary component records were included, and from 2003 to 2018, the average of 2 days of dietary data was used. The two dietary recall data collections were conducted separately: the first was carried out at the Mobile Examination Center, while the second was collected via telephone 3 to 10 days later. This approach helps provide a more comprehensive evaluation of each participant’s dietary habits. The calculation of the DII requires 45 dietary components; however, due to limitations in NHANES data collection, this study included 28 dietary components for DII calculation, which are: alcohol, vitamin B12, vitamin B6, *β*-carotene, caffeine, carbohydrate, cholesterol, energy, total fat, fiber, folic acid, iron, magnesium, monounsaturated fatty acid, niacin, n-3 fatty acid, n-6 fatty acid, protein, polyunsaturated fatty acid, riboflavin, saturated fat, selenium, thiamine, vitamin A, vitamin C, vitamin D, vitamin E, and zinc. Previous studies have shown that using fewer than 30 dietary components for DII calculation does not significantly affect its accuracy (6, 12). Even a DII calculation that incorporates 28 dietary components has a strong predictive ability for high CRP levels [OR 1.10 (1.02–1.19)] (6). The DII calculation formula is as follows:


$$Z\;\mathrm{score}=\frac{\mathrm{daily}\;\mathrm{mean}\;\mathrm{intake}-\mathrm{global}\;\mathrm{daily}\;\mathrm{mean}\;\mathrm{intake}}{\mathrm{standard}\;\mathrm{deviation}}$$

Convert the *Z*-score to a percentile score and then standardize it


$$Z\;{\mathrm{score}}^{\prime }=\left(Z\;\mathrm{score percentile score}\right)\times 2-1$$

$$DII=\sum \left(Z\;{\mathrm{score}}^{\prime }\times \mathrm{inflammation effect score}\right)$$

### 2.3 Outcome definition

The data of CHD was collected from the NHANES interview, where each question was standardized and administered by trained professionals. CHD was defined as “Has a doctor or other health professional ever told you that you had coronary heart disease? (MCQ160C).”

### 2.4 Covariates

The following variables were extracted from the NHANES: age, sex, race, education level, smoking, hypertension, diabetes, body mass index (BMI), glomerular filtration rate (GFR), total cholesterol, triglycerides, high-density lipoprotein (HDL), low-density lipoprotein (LDL), and hemoglobin A1c. Smoking was defined as having smoked more than 100 cigarettes in one’s lifetime. Biochemical parameters were evaluated using a standardized methodology detailed in the NHANES Laboratory/Medical Technologist Procedure Manual (CDC: NHANES Laboratory/Medical Technologists Procedures Manual, Atlanta, GA, CDC, 2001).

The triglyceride-glucose index (TyG) is defined as ln[triglycerides (mg/dL)/fasting glucose (mg/dL)/2]. The Waist-to-Height Ratio (WHtR) is defined as waist circumference (cm)/height (cm). The Framingham score, first proposed in 2008 to assess cardiovascular disease risk, was updated in 2015^1^ (13). The formula for the visceral adiposity index (VAI) is as follows:


$$VAI=\frac{\mathrm{waist}\;\mathrm{circumference}}{\left(39.68+1.88\times BMI\right)}\times \frac{TG}{1.03}\times \frac{1.31}{HDL}\;\left(\mathrm{Male}\right)$$

$$VAI=\frac{\mathrm{waist}\;\mathrm{circumference}}{\left(36.58+1.89\times BMI\right)}\times \frac{TG}{0.81}\times \frac{1.52}{HDL}\;\left(\mathrm{Female}\right)$$

### 2.5 Statistical methods

Due to the complexity of NHANES sampling, weighted regression models were used with WTDRD1 and WTDR2D as weights. All participants were divided into three groups based on the tertiles of the DII. Normally distributed continuous data were expressed as means ± standard deviations, and differences between the three groups were examined using one-way ANOVA. Non-normally distributed continuous data were expressed as medians (Q1, Q3) and compared using the Kruskal-Wallis H test. For continuous variables, effect sizes were calculated using Cohen’s F, and *post hoc* comparisons were performed using the Newman–Keuls method. Categorical data were presented as counts and percentages and were compared using the chi-squared test, with Cramér’s V employed to assess effect sizes and calculating pairwise comparisons between pairs of proportions with bonferroni correction for multiple testing. The relationship between DII and traditional cardiovascular risk factors was assessed using restricted cubic splines (RCS). The association between DII and CHD was explored using multivariable logistic regression models, with DII as both continuous and categorical variable to evaluate the robustness of the results.

In exploring the mediating factors in the association between DII and CHD, two models were constructed. The first is the mediator model, using generalized linear models (GLM) to fit the Gaussian distribution (linear regression with an identity link function) between DII and potential mediators, while controlling for potential confounders. The second is the outcome model, which uses both GLM and logistic regression models, incorporating both exposure factors and mediators. To ensure the reliability of the results, Bootstrap resampling was performed 1,000 time (14). Further subgroup analyses were conducted based on age, sex, cholesterol levels, and Framingham risk scores to assess whether there are interaction effects between DII and these factors. The mean decrease in Gini (MDG) is a commonly used method for measuring feature importance in random forest models, reflecting the contribution of each dietary component to the model’s predictive performance.

Statistical significance was set at *p* < 0.05. All analyses were performed using R version 4.2.0 (R Foundation for Statistical Computing, Vienna, Austria). The analysis mainly used the ‘survey’, ‘plotRCS’, ‘mediation’, and ‘randomForest’ packages.

## 3 Results

### 3.1 Characteristics of the study population

A total of 43,842 participants were included in this study, with a mean age of 49.81 ± 18.10 years, of whom 20,793 (47.4%) were male. Among the participants, 15,310 (34.9%) had hypertension, and 5,289(12.1%) had diabetes. The DII score was 1.33 (0.11, 2.40). Participants were divided into three groups based on the DII tertiles, with 14,614 individuals in each group. Significant differences were observed between the groups in terms of age, sex, race, education level, hypertension, diabetes, CHD, BMI, GFR, total cholesterol and HDL (all *p* < 0.05). The characteristics and effect size data of the three groups were shown in Table 1. Pairwise comparisons between the three groups were performed (Supplementary Table S1). Further comparisons of dietary components between the three groups revealed significant differences across all 28 dietary components included in this study (all *p* < 0.001) (Supplementary Tables S2, S3).

**Table 1:** The characteristics of participants from the 1999–2018 NHANES.

Variate	Overall (*N* = 43,842)	DII Tertile 1 (*N* = 14,614)	DII Tertile 2 (*N* = 14,614)	DII Tertile 3 (*N* = 14,614)	*p*	Effect size^*^
Age, years	49.81 ± 18.10	48.66 ± 17.57	49.54 ± 18.14	51.22 ± 18.49	<0.001	0.057
Sex, male	20,793 (47.4%)	8,912 (61.0%)	6,875 (47.0%)	5,006 (34.3%)	<0.001	0.219
Race					<0.001	0.079
Mexican American	7,595 (17.3%)	2,356(16.1%)	2,656 (18.2%)	2,583 (17.7%)		
Other Hispanic	3,455 (7.9%)	978 (6.7%)	1,093 (7.5%)	1,384 (9.5%)		
Non-Hispanic White	20,134 (45.9%)	7,485 (51.2%)	6,729 (46.0%)	5,920 (40.5%)		
Non-Hispanic Black	9,147 (20.9%)	2,488 (17.0%)	2,977 (20.4%)	3,682 (25.2%)		
Other Race	3,511 (8.0%)	1,307 (8.9%)	1,159 (7.9%)	1,045 (7.2%)		
Education level					<0.001	0.138
No high school diploma	4,932 (11.2%)	1,059(7.2%)	1,655 (11.3%)	2,218 (15.2%)		
Some high school	6,399 (14.6%)	1,694 (11.6%)	2072 (14.2%)	2,633 (18.0%)		
High school graduate	10,159 (23.2%)	3,047 (20.8%)	3,414 (23.4%)	3,698 (25.3%)		
Some college/associate degree	12,556 (28.6%)	4,261 (29.2%)	4,260 (29.2%)	4,035 (27.6%)		
Bachelor’s degree or higher	9,747 (22.2%)	4,545 (31.1%)	3,188 (21.8%)	2014 (13.8%)		
Smoking	19,991 (45.6%)	6,639 (45.4%)	6,667 (45.6%)	6,685 (45.7%)	0.859	0.003
Hypertension	15,310 (34.9%)	4,588 (31.4%)	5,002 (34.2%)	5,720 (39.1%)	<0.001	0.068
Diabetes	5,289 (12.1%)	1,421 (9.7%)	1729 (11.8%)	2,139 (14.6%)	<0.001	0.062
CHD	1892 (4.3%)	580 (4.0%)	613 (4.2%)	699 (4.8%)	0.002	0.017
BMI, kg/m^2^	29.06 ± 6.79	28.40 ± 6.43	29.15 ± 6.76	29.64 ± 7.12	<0.001	0.076
GFR, mL/min/1.73 m^2^	94.49 ± 24.96	95.85 ± 23.17	94.85 ± 24.94	92.72 ± 26.58	<0.001	0.051
Cholesterol, mmol/L	4.99 (4.32, 5.74)	4.99 (4.32, 5.72)	5.02 (4.34, 5.77)	4.97 (4.29, 5.72)	0.001	0.018
HDL, mmol/L	1.37 ± 0.42	1.38 ± 0.42	1.37 ± 0.42	1.36 ± 0.41	<0.001	0.022
LDL, mmol/L	3.00 ± 0.93	2.98 ± 0.90	3.01 ± 0.95	3.01 ± 0.95	0.073	0.016
HbA1c, %	7.0 (6.1, 8.0)	7.0 (6.1, 7.8)	7.0 (6.2, 7.7)	7.0 (6.1, 8.0)	0.857	0.026
TyG	8.66 ± 0.68	8.62 ± 0.69	8.66 ± 0.68	8.69 ± 0.67	<0.001	0.042
WHtR	0.59 ± 0.10	0.58 ± 0.09	0.59 ± 0.10	0.61 ± 0.10	<0.001	0.12
VAI	0.044 (0.026, 0.077)	0.041 (0.024, 0.074)	0.044 (0.026, 0.076)	0.047 (0.028, 0.081)	<0.001	0.03
Framingham score	12 (5, 16)	11 (4, 15)	12 (5, 16)	13 (6, 17)	<0.001	0.094

^*^For continuous variables, effect sizes were calculated using Cohen’s f, and for categorical variables, effect sizes were calculated using Cramér’s V. DII, dietary inflammatory index; CHD, coronary heart disease; BMI: body mass index; GFR, glomerular filtration rate; HDL, high-density lipoprotein; LDL, low-density lipoprotein; TyG, triglyceride-glucose index; WHtR, waist-to-height ratio; VAI, visceral adiposity index.

### 3.2 Association between DII and CHD, and risk factors

**Figure 2:**
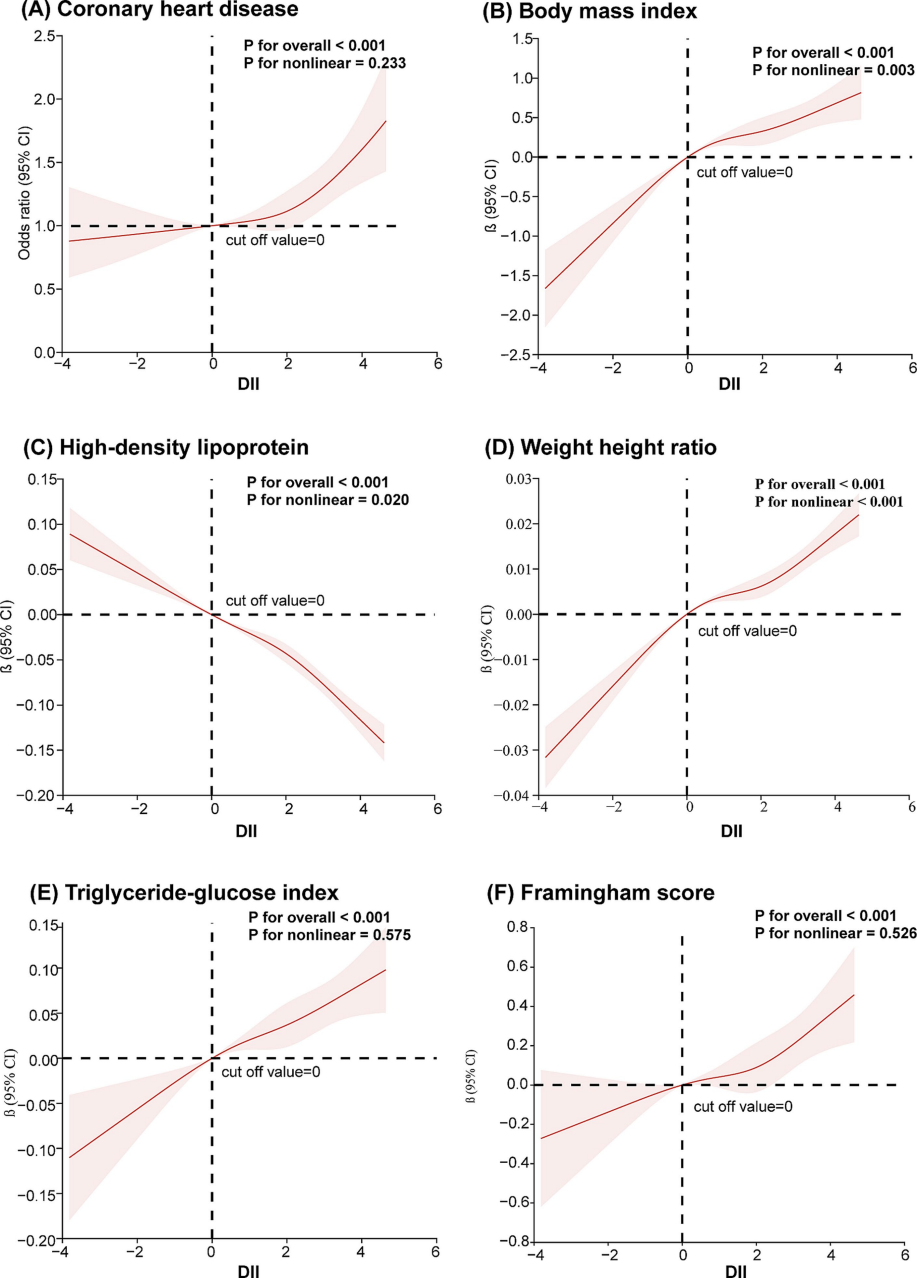
Restricted cubic spline analysis of the association between dietary inflammation index and coronary heart disease and its risk factors **(A)** coronary heart disease; **(B)** body mass index; **(C)** high-density lipoprotein; **(D)** waist-to-height ratio; **(E)** triglyceride and glucose index; **(F)** Framingham score. A restricted cubic spline model was calculated after adjusting for age, sex, race and education level.

After adjusting for age, sex, race, and education level, the relationship between DII, CHD, and common risk factors was analyzed using RCS. The knots between 3 and 7 were tested, and the model with the lowest Akaike information criterion value was selected for the RCS analysis. As shown in Figure 2A, there appears to be a J-shaped curve between DII and CHD, with the risk of CHD increasing at an accelerating rate as DII rises. The results indicated a positive correlation between DII and WHtR, BMI, TyG, and the Framingham score (Figures 2B,D–F). DII was negatively correlated with HDL (Figure 2C).

### 3.3 The association between DII and CHD

For confounder adjustment, multivariable logistic regression was used to analyze the relationship between DII and CHD. DII was analyzed as a continuous variable. Based on previous literature (15) and the covariates included in this study, Model 1 did not adjust for any covariates; Model 2 adjusted for age, sex, race, and education; Model 3 adjusted for hypertension, diabetes, smoking, BMI, GFR and total cholesterol in addition to the covariates in Model 2. In the unadjusted model, a higher DII was significantly associated with an increased risk of CHD [OR: 1.057 (1.026–1.089), *p* < 0.001]. In Model 2, the association between DII and CHD remained significant [OR: 1.065 (1.031–1.101), *p* < 0.001]. In Model 3, the correlation between DII and CHD continued to exist [OR: 1.049 (1.012–1.087), *p* = 0.008]. When DII was categorized into lower, middle, and higher DII groups, the analysis results remained consistent [OR: 1.095 (1.024–1.171), *p* = 0.008] (Table 2).

**Table 2:** The association between DII and CHD in different logistics models.

	DII as a continuous variable^a^			DII as a nominal variable^b^		
	OR	95% CI	*p*	OR	95% CI	*p*
Model 1	1.057	1.026–1.089	< 0.001	1.104	1.043–1.168	< 0.001
Model 2	1.065	1.031–1.101	< 0.001	1.116	1.050–1.188	< 0.001
Model 3	1.049	1.012–1.087	0.008	1.095	1.024–1.171	0.008

Model 1: Crude. Model 2: Adjusted for Model 1, age, sex, race and education. Model 3: Adjusted for Model 2, hypertension, diabetes, smoking, body mass index, glomerular filtration rate, and total cholesterol. OR, odds ratio; CI confidence interval.

a OR was examined by per 1-unit increase of DII.

b divided into lower, middle, and higher DII groups based on the tertiles of the DII distribution.

### 3.4 Mediators between DII and CHD

**Figure 3:**
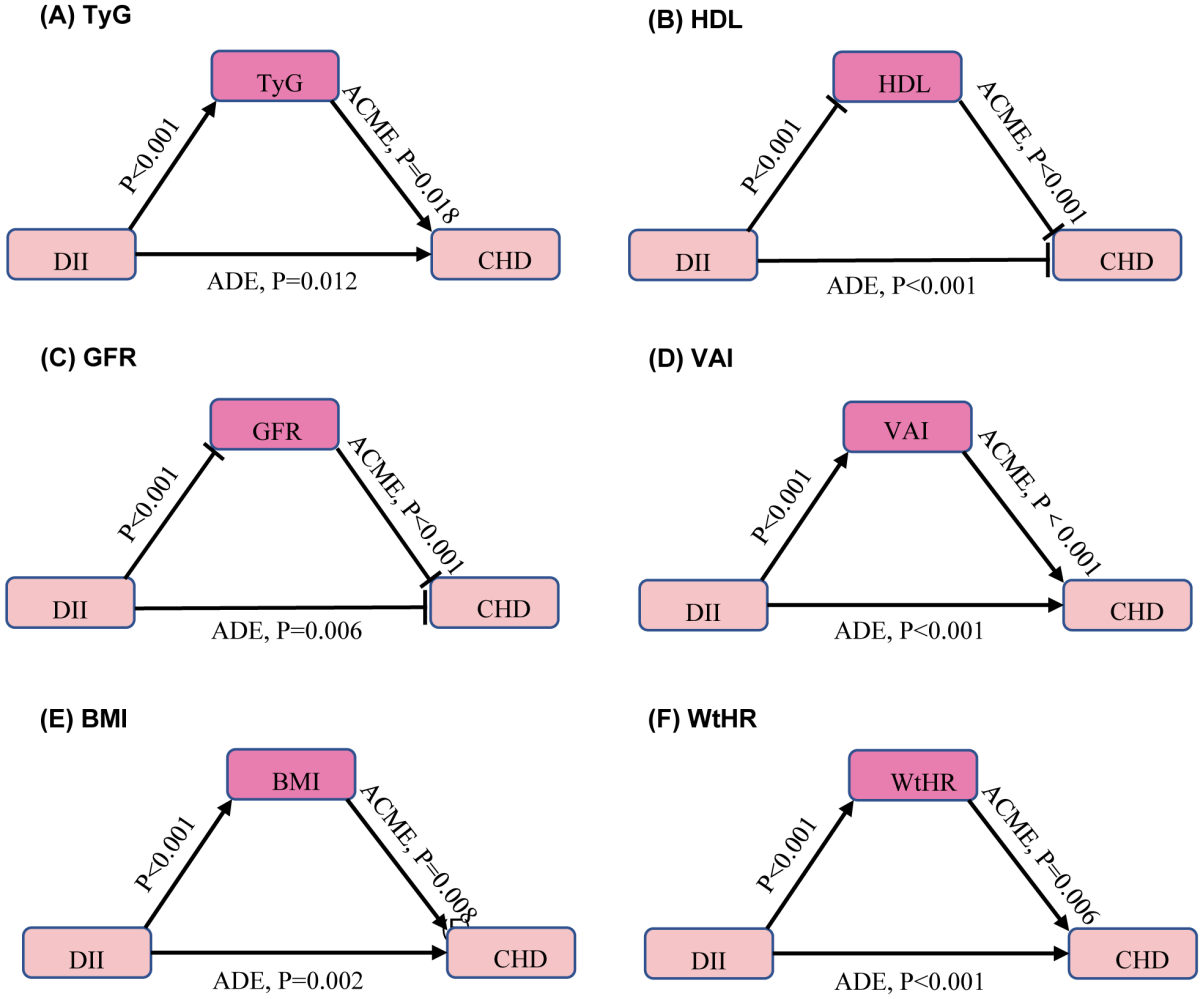
Mediators of the association between dietary inflammation index and coronary heart disease. Arrows and rounded heads indicate promotion and inhibition, respectively. The model was calculated after adjusting for age, sex, race and education level. ACME, average causal mediation effects; ADE, average direct effects. TyG, triglyceride-glucose index; HDL, high-density lipoprotein cholesterol; GFR, glomerular filtration rate; VAI, visceral adiposity index; BMI, body mass index; WHtR, waist-to-height ratio. **(A)** TyG, **(B)** HDL, **(C)** GFR, **(D)** VAI, **(E)** BMI, **(F)** WtHR.

Given the correlation between DII and traditional CHD risk factors, further analysis of the mediators between DII and CHD was conducted after adjusting for age, sex, race, and education level. The average direct effect (ADE) represents the direct effect of DII on the outcome variable without mediation, while the average causal mediation effect (ACME) represents the indirect effect of DII on the outcome variable through mediators. The results indicated that DII indirectly increases the risk of CHD by elevating TyG, VAI, BMI, and WHtR. Additionally, DII was found to increase the risk of CHD by lowering HDL and GFR (Figure 3). However, since this study is cross-sectional, causality still needs to be confirmed through further longitudinal research.

### 3.5 The impact of DII on CHD in different subgroups

**Figure 4:**
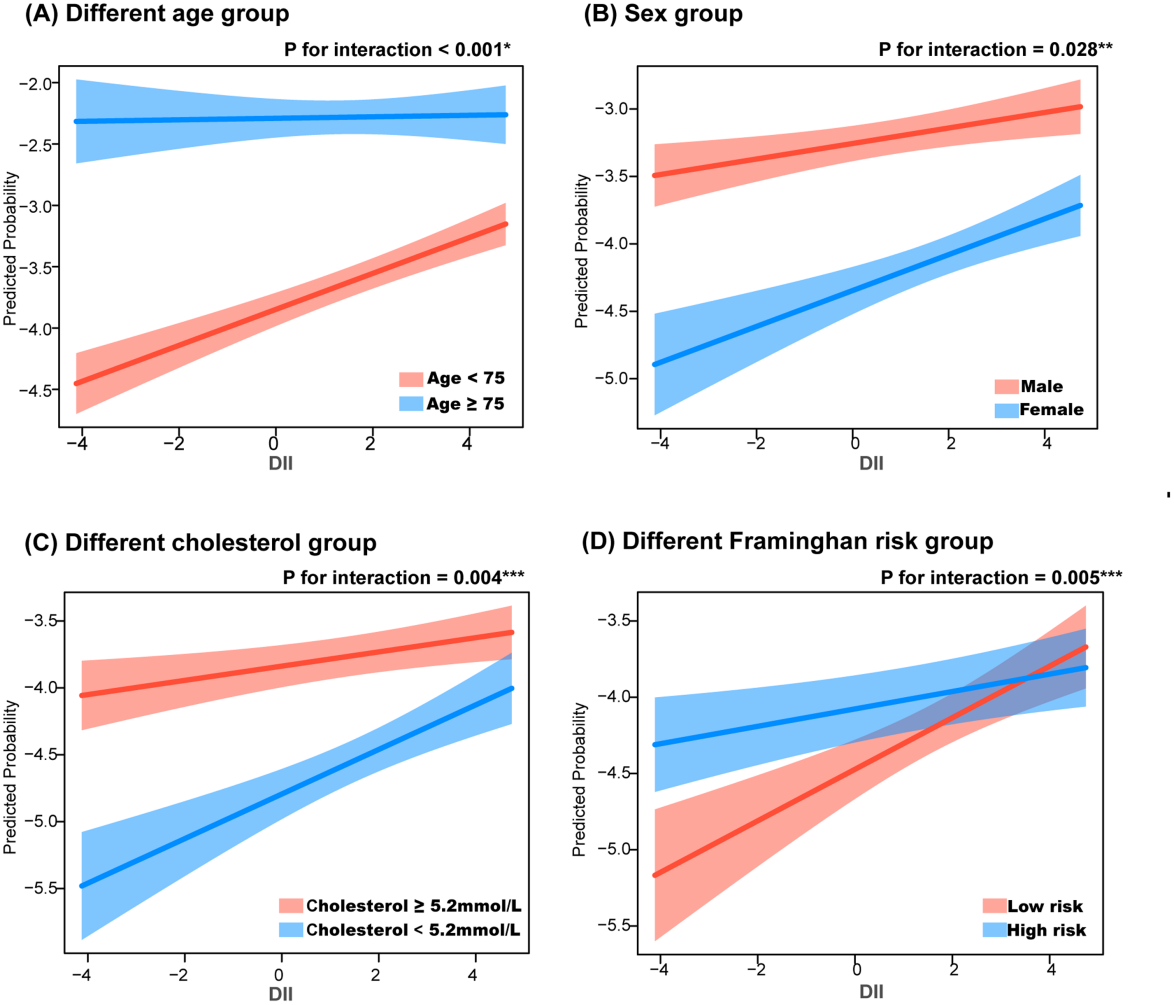
Subgroup analysis in the relationship between dietary inflammation index and coronary heart disease. ^*^, adjusting for sex, race and education level; ^**^, adjusting for age, race and education level; ^***^, adjusting for age, sex, race and education level. **(A)** Different age group, **(B)** Sex group, **(C)** Different cholesterol group, **(D)** Different framinghan risk group.

To further explore the role of DII in different populations, subgroup analyses were performed based on age (≥75 years vs. <75 years), sex, cholesterol levels (≥5.2 mmol/L vs. <5.2 mmol/L), and Framingham risk score. After adjusting for sex, race, and education level, the subgroup aged <75 years showed higher sensitivity to DII (*p* for interaction <0.001). When adjusted for age, race, and education level, a stronger association between DII and CHD was observed in females (*p* for interaction = 0.028). In the Framingham risk groups, individuals with a 10-year heart disease risk >10% were classified as high-risk (men ≥12 points, women ≥20 points). Further analysis showed that, after adjusting for age, sex, race, and education level, individuals with low cholesterol levels (*p* for interaction = 0.004) and those in the Framingham low-risk group (*p* for interaction = 0.005) were more sensitive to DII (Figure 4).

### 3.6 Contribution of dietary components to CHD

**Figure 5:**
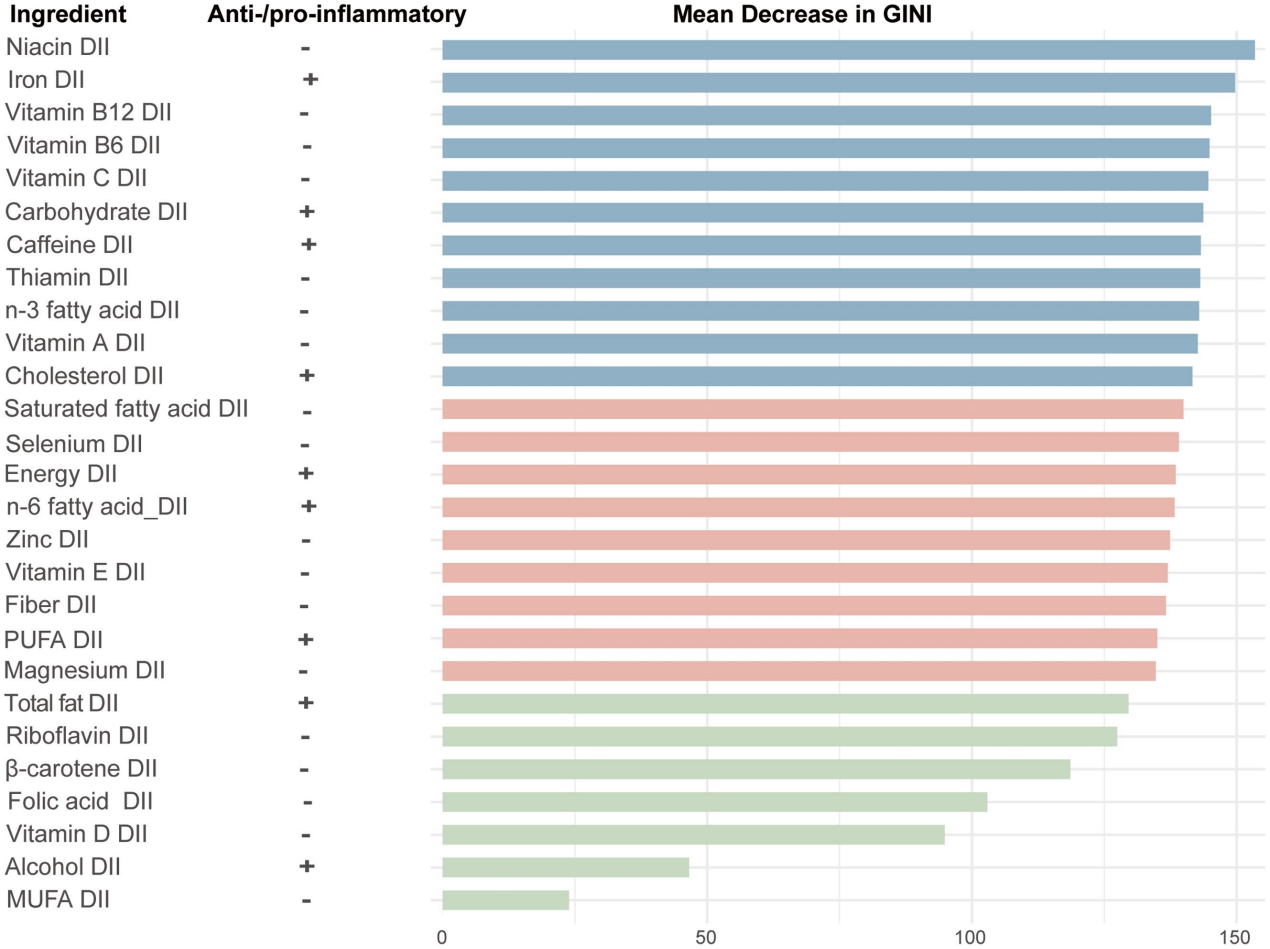
The importance ranking of 28 dietary components in the dietary inflammation index for CHD using random forest. +, pro-inflammatory; −, anti-inflammatory; GINI, gini index; PUFA, polyunsaturated fatty acids; MUFA, monounsaturated fatty acids.

To assess the contribution of dietary components to CHD, we used a random forest model to calculate the MDG for each dietary component. The larger the MDG value, the greater the contribution of that dietary component to the model’s performance. The results showed that the top 5 dietary components contributing the most to CHD were carbohydrate, vitamin C, iron, vitamin A and caffeine, while the bottom 3 components were vitamin D, alcohol, and monounsaturated fatty acids (Figure 5).

## 4 Discussion

This study, which included 43,842 participants from the NHANES between 1999 and 2018, showed the association between the DII and CHD, and explored the potential mediating factors and interactions. The results showed that: (1) After adjusting for multiple variables, DII remained independently associated with CHD. (2) DII may be associated with CHD through its impact on factors such as TyG, VAI, BMI, WHtR, HDL and GFR. (3) The association between DII and CHD was more sensitive in individuals aged <75 years, females, those with low cholesterol levels, and those in the low-risk Framingham score group. (4) Among the 28 dietary components analyzed in this study, carbohydrate, vitamin C and iron were found to have the greatest association on CHD.

CHD is a chronic inflammatory disease influenced by various factors. The DII is an inflammation indicator based on dietary components, and studies have shown that DII is correlated with multiple inflammatory markers in the body (6–8, 16). Previous research has also confirmed the association between DII and CHD (9). In this study, despite adjusting for age, sex, race, education level, and traditional cardiovascular risk factors, DII remained independently associated with CHD. Prior studies showed that higher DII in CHD patients is associated with significantly reduced plaque stability, indicating that anti-inflammatory diets may play an important protective role in the pathogenesis of CHD (17).

In this study, TyG, VAI, BMI, WHtR, HDL, and GFR may serve as mediators in the relationship between DII and CHD. TyG index is a well-established indicator of insulin resistance and has been shown to effectively predict the risk of various cardiovascular diseases, including CHD, atherosclerosis, and stroke (18, 19). Pro-inflammatory diets elevate circulating inflammatory cytokines (e.g., TNF-*α*, IL-6), which impair insulin signaling in adipose tissue, muscle, and liver. Chronic insulin resistance directly damages vascular endothelial cells and smooth muscle cells, promoting atherosclerotic plaque formation (20). Additionally, excessive reactive oxygen species and harmful glycation products generated by hyperglycemia contribute to chronic inflammation, further amplifying the risk of CHD (21). Shu et al. (22) reported that DII was positively correlated with fasting glucose, fasting insulin, and the homeostasis model assessment of insulin resistance. Furthermore, a cohort study further suggested that the relationship between pro-inflammatory diets and nonfatal cardiovascular diseases was partially mediated by TyG (23).

VAI, BMI, and WHtR are indicators of visceral adiposity and obesity, and they exhibit strong associations with CHD and all-cause mortality (24, 25). Pro-inflammatory diets, such as high-fat diets, activate the pro-inflammatory factor NF-κB by inducing endoplasmic reticulum (ER) stress and promoting the interaction of TLR4 and TLR2. Oxidative stress in the ER plays a critical role in initiating inflammation and metabolic disorders, further contributing to visceral fat accumulation and obesity (26). Moreover, dietary inflammation levels may influence gut microbiota composition. Anti-inflammatory diets have been shown to lower the proportion of Gram-negative bacteria in the gut, strengthen intestinal barrier function, and reduce endotoxin levels, thereby mitigating metabolic and inflammatory responses (27).

Moreover, pro-inflammatory diets promote inflammatory markers (TNF-*α* and NF-κB) and oxidative stress markers (elevated NADPH oxidase activity) in the renal cortex, inducing mesangial cell proliferation and the progression of proteinuria, ultimately leading to a decline in GFR (28). Chronic kidney disease is often associated with lipid metabolism disorders, and the accumulation of toxins in the body can directly damage endothelial cells, promote platelet adhesion, and contribute to the formation of atherosclerotic plaques (29). Due to incomplete and methodologically inconsistent CRP/hsCRP measurements in NHANES (1999–2018), CRP was not analyzed as a mediator in this study.

Subgroup analyses were conducted to examine the relationship between DII and CHD across different CHD risk factors, including age, sex, total cholesterol levels, and Framingham risk score, to evaluate the robustness of the findings. This study found that elevated DII may be more strongly associated with CHD in individuals aged <75 years, females, those with low cholesterol levels, and individuals at low risk group according to the Framingham score. A prospective cohort study involving 155,724 participants, with approximately 10 years of follow-up, revealed that poor dietary quality was more strongly associated with cardiovascular disease in women than in men, and DII was also linked to a higher risk of all-cause mortality in female CHD patients (11, 30). From Figure 4D, it can be observed that when DII is at a low level, the CHD risk in the low-risk group is significantly lower compared to the high-risk group. As DII increases, the CHD risk in the low-risk group rises rapidly, ultimately approaching that of the high-risk group. This suggests that the impact of DII is more sensitive in the low-risk population than in the high-risk group. One possible explanation is that individuals in the low-risk group lack other strong risk factors, making the inflammatory response triggered by diet more prominent. Additionally, high-risk individuals may have access to more health education and lifestyle interventions, thereby reducing their risk of CHD. Finally, DII may exert a greater effect in the low-risk group through interactions with other metabolic and inflammatory markers. Despite adjusting for general demographic characteristics (sex, age, race, and education level), potential confounding factors may still exist, requiring further validation through prospective cohort studies and mechanistic research.

Calculating the DII requires the collection of 45 different dietary components, which poses a challenge for both doctors and patients in clinical practice. This increases the complexity of assessment due to individual differences, dietary habits, and measurement errors. In this study, we used the random forest method to rank the contribution of dietary components to CHD risk based on their DII. However, this is only a preliminary exploration, and further research is needed to assess its feasibility and accuracy.

Our study found that carbohydrate, vitamin C and iron were the three components with the greatest DII contribution to CHD. Carbohydrates are the primary source of energy in the diet. A study involving over 10,000 participants, followed for more than a decade, found a significant association between carbohydrate intake and major adverse cardiovascular events (HR: 1.35; 95%CI: 1.07–1.71; *p*-trend = 0.001) (31). Another study from 18 countries showed a non-linear relationship between carbohydrate intake and cardiovascular disease mortality (32). Vitamin C is a well-known antioxidant. A research has shown that the intake of vitamin C is negatively correlated with the carotid intima-media thickness in patients with CHD (*r* = −0.113, *p* = 0.001) (33). Additionally, vitamin C intake is positively correlated with plaque stability in these patients and may exert its effects by reducing IL-6 and TNF-*α*, thereby inhibiting the inflammatory response in atherosclerosis (34). Iron’s DII ranked third in contributing to CHD risk. Iron intake has been positively correlated with CRP levels (*p* trend = 0.03) (35). A meta-analysis of 6 prospective studies indicated that individuals with higher heme iron intake had a 31% increased risk of CHD (36). This suggests that iron intake, particularly heme iron, may need to be controlled to reduce inflammation.

Interestingly, alcohol, typically considered a risk factor for CHD, ranked second to last in DII contribution. This may be related to the relatively low alcohol intake in the study population compared to the upper limits recommended in current guidelines. In the United States, the recommended alcohol intake limit is 196 g per week for men and 98 g per week for women (37). A J-shaped curve relationship exists between alcohol consumption and CHD (38). Recent research has found that reducing alcohol consumption can help consistently lower cardiovascular disease risk, although a clear threshold for alcohol intake remains undefined (39).

### 4.1 Limitations

This study has several limitations. First, since NHANES is a cross-sectional survey, this study cannot determine a causal relationship between DII and CHD risk, but only an association between the two. Future studies with longitudinal designs are needed to better evaluate the impact of DII on CHD risk. Second, dietary data were collected using recall methods, which may introduce subjective bias and may not accurately reflect long-term daily dietary intake. However, studies have shown a strong correlation between dietary data obtained through food records and 24-h recalls (40). Moreover, 24-h recall methods have been widely used in dietary intervention trials and national surveys (40). Third, the outcome variable in this study was based on self-reported questionnaires, which are subject to recall bias, information bias, and potential misclassification of exposure. Although NHANES implements multiple measures to minimize recall and information bias during data collection, the possibility of data quality issues remains. Lastly, although we adjusted for potential confounding factors, the influence of other unmeasured confounders on the results cannot be entirely excluded.

## 5 Conclusion

Higher DII is independently associated with an increased risk of CHD, potentially through pathways involving metabolism, lipid levels, and kidney function. The impact of DII on CHD is more sensitive in individuals with low traditional risk. These findings provide new evidence for the role of dietary interventions in reducing CHD incidence and lay the groundwork for future cohort studies and mechanistic investigations.

## Data Availability

Publicly available datasets were analyzed in this study. This data can be found at: https://wwwn.cdc.gov/nchs/nhanes/.

## Footnotes

^1^ https://www.thecalculator.co/health/Framingham-Risk-Score-Calculator-for-Coronary-Heart-Disease-745.html
