# Supplementary material for: The relationship between dietary inflammatory index in adults and coronary heart disease: from NHANES 1999–2018
Source: Front Nutr. 2025 Mar 21;12:1564580. doi: 10.3389/fnut.2025.1564580 (PMC11970130; doi:10.3389/fnut.2025.1564580)
Supplement: Supplementary file 1 [file Table_1.docx]

Supplementary Material

# Supplementary Tables

Supplement Table S1. Post-hoc test results in the characteristics of participants across different DII subgroups

| Variate | Tertile 1 VS Tertile 2 | Tertile 1 VS Tertile 3 | Tertile 2 VS Tertile 3 |
| --- | --- | --- | --- |
| Age, years | 0.813 (P < 0.001) | 2.475 (P < 0.001) | 1.662 (P <0 .001) |
| Sex, male | P < 0.001 | P < 0.001 | P < 0.001 |
| Race |  |  |  |
| Mexican American | P < 0.001 | P = 0.001 | P = 0.816 |
| Other Hispanic | P = 0.028 | P < 0.001 | P < 0.001 |
| Non-Hispanic White | P < 0.001 | P < 0.001 | P < 0.001 |
| Non-Hispanic Black | P < 0.001 | P < 0.001 | P < 0.001 |
| Other Race | P = 0.006 | P < 0.001 | P = 0.037 |
| Education level |  |  |  |
| No high school diploma | P < 0.001 | P < 0.001 | P < 0.001 |
| Some high school | P < 0.001 | P < 0.001 | P < 0.001 |
| High school graduate | P < 0.001 | P < 0.001 | P < 0.001 |
| Some college/associate degree | P = 0.998 | P =0.011 | P = 0.009 |
| Bachelor's degree or higher | P < 0.001 | P < 0.001 | P < 0.001 |
| Smoking | P > 0.999 | P > 0.999 | P > 0.999 |
| Hypertension | P < 0.001 | P < 0.001 | P < 0.001 |
| Diabetes | P < 0.001 | P < 0.001 | P < 0.001 |
| CHD | P > 0.999 | P = 0.002 | P = 0.049 |
| BMI, kg/m^2^ | 0.749 (P < 0.001) | 1.248 (P < 0.001) | 0.499 (P < 0.001) |
| GFR, mL/min/1.73 m^2^ | 0.913(P = 0.002) | 3.047 (P < 0.001) | 2.134 (P < 0.001) |
| Cholesterol, mmol/L | 0.043 (P=0.003) | 0.001 (P < 0.001) | 0.042 (P < 0.001) |
| HDL, mmol/L | 0.200 (P = 0.300) | 0.832 (P < 0.001) | 0.632 (P = 0.001) |
| LDL, mmol/L | 1.307 (P = 0.097) | 1.073 (P = 0.908) | 0.233 (P = 0.712) |
| HbA1c, % | 0.088 (P = 0.499) | 0.120 (P = 0.598) | 0.032 (P = 0.750) |
| TyG | 0.039 (P < 0.001) | 0.070 (P < 0.001) | 0.031 (P = 0.007) |
| WHtR | 0.016 (P < 0.001) | 0.029 (P < 0.001) | 0.013 (P < 0.001) |
| VAI | 0.002 (P = 0.016) | 0.006 (P < 0.001) | 0.004 (P < 0.001) |
| Framingham score | 0.721 (P < 0.001) | 1.773 (P < 0.001) | 1.052 (P < 0.001) |

Note: For continuous variables, the Newman-Keuls method was performed as the post-hoc testing, reporting the absolute value of the differences and p-values. For categorical variables, pairwise comparisons between pairs of proportions with Bonferroni correction for multiple testing were conducted, and adjusted p-values were recorded.

Supplement Table S2. Comparison of various dietary components across different DII groups

| Food parameter | Overall | Tertile 1 | Tertile 2 | Tertile 3 | P | Effect size* |
| --- | --- | --- | --- | --- | --- | --- |
| Alcohol, g | 0.00 (0.00, 3.05) | 0.00 (0.00, 14.05) | 0.00 (0.00, 3.70) | 0.00 (0.00, 0.00) | <0.001 | 0.150 |
| vitamin B12, μg | 3.90 (2.40, 6.11) | 5.99 (4.06, 8.71) | 4.11 (2.76, 5.80) | 2.49 (1.57, 3.62) | <0.001 | 0.296 |
| vitamin B6, mg | 1.74 (1.23, 2.41) | 2.60 (2.10, 3.30) | 1.75 (1.41, 2.14) | 1.12 (0.83, 1.43) | <0.001 | 0.692 |
| Beta-carotene, μg | 1048(414, 2672) | 2702 (1071, 5572) | 1120 (500, 2476) | 507 (233, 1200) | <0.001 | 0.409 |
| Caffeine, g | 602 (1.44, 2022) | 1429 (0.55, 4294) | 653(0.41, 1921) | 362 (69, 976) | <0.001 | 0.312 |
| Carbohydrate, g | 232 (173, 307) | 303 (238, 383) | 234 (187, 292) | 174 (131, 226) | <0.001 | 0.578 |
| Cholesterol, mg | 239 (148, 375) | 311 (202, 481) | 249 (164, 377) | 172 (107, 278) | <0.001 | 0.347 |
| Energy, kcal | 1905  (1444, 2482) | 2551  (2048, 3170) | 1934  (1589, 2348) | 1381  (1082, 1741) | <0.001 | 0.734 |
| Total fat, g | 0.07 (0.05, 0.10) | 0.10 (0.07, 0.13) | 0.07 (0.05, 0.09) | 0.05 (0.04, 0.07) | <0.001 | 0.605 |
| Fiber, g | 0.01 (0.01, 0.02) | 0.02 (0.02, 0.03) | 0.01 (0.01, 0.02) | 0.01 (0.01, 0.01) | <0.001 | 0.696 |
| Folic acid, μg | 139 (81, 227) | 214 (119, 344) | 152 (93, 228) | 101 (61, 151) | <0.001 | 0.433 |
| Iron, mg | 13.19  (9.58, 18.17) | 19.00  (14.93, 24.76) | 13.43  (10.73, 16.76) | 9.05  (6.82, 11.52) | <0.001 | 0.727 |
| Magnesium, mg | 263 (195, 348) | 380 (317, 463) | 264 (222, 309) | 179 (140, 220) | <0.001 | 0.973 |
| MUFA, g | 0.03 (0.02, 0.04) | 0.03 (0.03, 0.05) | 0.03 (0.02, 0.03) | 0.02 (0.01, 0.02) | <0.001 | 0.572 |
| Niacin, mg | 21.73  (15.68, 29.44) | 30.93  (24.53, 39.11) | 22.07  (17.68, 27.20) | 14.92  (11.26, 19.05) | <0.001 | 0.713 |
| n-3 fatty acid, g | 1.46  (0.96, 2.15) | 2.12  (1.52, 2.94) | 1.49  (1.07, 2.03) | 0.98  (0.66, 1.40) | <0.001 | 0.549 |
| n-6 fatty acid, g | 13.53  (8.99, 19.49) | 19.30  (13.88, 26.03) | 13.77  (9.88, 18.48) | 9.31  (6.28, 13.03) | <0.001 | 0.571 |
| Protein, g | 71.7 (50.5, 97.3) | 100 (78.3, 130) | 72.7 (56.4, 90.2) | 46.4 (33.5, 60.8) | <0.001 | 0.761 |
| PUFA, g | 0.02 (0.01, 0.02) | 0.02 (0.02, 0.03) | 0.02 (0.01, 0.02) | 0.01 (0.01, 0.01) | <0.001 | 0.585 |
| Riboflavin, mg | 1.84 (1.32, 2.50) | 2.61 (2.06, 3.33) | 1.87 (1.48, 2.31) | 1.26 (0.95, 1.62) | <0.001 | 0.689 |
| Saturated fat, g | 0.02 (0.02, 0.03) | 0.03 (0.02, 0.04) | 0.02 (0.02, 0.03) | 0.02 (0.01, 0.02) | <0.001 | 0.488 |
| Selenium, μg | 99 (72, 133) | 135 (104, 175) | 102. (80, 127) | 71 (53, 91) | <0.001 | 0.637 |
| Thiamin, mg | 1.43 (1.04, 1.94) | 2.04 (1.61, 2.58) | 1.46 (1.16, 1.80) | 1.00 (0.75, 1.28) | <0.001 | 0.734 |
| Vitamin A, RE | 517 (316, 802) | 865 (619, 1203) | 516 (361, 703) | 304 (192, 441) | <0.001 | 0.448 |
| Vitamin C, mg | 66.10  (30.85, 120.35) | 116.40  (67.44, 179.63) | 64.20  (33.95, 110.01) | 34.70  (16.25, 68.14) | <0.001 | 0.480 |
| Vitamin D, μg | 3.45 (1.85, 5.95) | 6.00 (3.60, 9.30) | 3.70 (2.15, 5.75) | 2.20 (1.10, 3.55) | <0.001 | 0.496 |
| Vitamin E, mg | 6.66 (4.54, 9.60) | 10.51 (8.10, 13.91) | 6.49 (4.98, 8.33) | 4.31 (3.08, 5.78) | <0.001 | 0.705 |
| Zinc, mg | 9.89  (7.03, 13.72) | 14.18  (11.17, 18.50) | 10.12  (7.94, 12.78) | 6.67  (5.00, 8.57) | <0.001 | 0.528 |

Note: * For continuous variables, effect sizes were calculated using Cohen's f.

Supplement Table S3. Post-hoc test results in dietary components across different DII groups

| Food parameter | Tertile 1 VS Tertile 2 | Tertile 1 VS Tertile 3 | Tertile 2 VS Tertile 3 |
| --- | --- | --- | --- |
| Alcohol, g | 4.242 (P < 0.001) | 8.391 (P < 0.001) | 4.149 (P < 0.001) |
| vitamin B12, μg | 2.701 (P < 0.001) | 4.562 (P < 0.001) | 1.861 (P < 0.001) |
| vitamin B6, mg | 1.012 (P < 0.001) | 1.701 (P < 0.001) | 0.689 (P < 0.001) |
| Beta-carotene, μg | 2323.939 (P < 0.001) | 3296.234 (P < 0.001) | 972.295 (P < 0.001) |
| Caffeine, g | 1633.601 (P < 0.001) | 2290.404 (P < 0.001) | 656.804 (P < 0.001) |
| Carbohydrate, g | 77.283 (P < 0.001) | 137.665 (P < 0.001) | 60.382 (P < 0.001) |
| Cholesterol, mg | 80.599 (P < 0.001) | 162.510 (P < 0.001) | 81.911 (P < 0.001) |
| Energy, kcal | 685.934 (P < 0.001) | 1255.803 (P < 0.001) | 569.869 (P < 0.001) |
| Total fat, g | 0.027 (P < 0.001) | 0.05 (P < 0.001) | 0.023 (P < 0.001) |
| Fiber, g | 0.008 (P < 0.001) | 0.013 (P < 0.001) | 0.005 (P < 0.001) |
| Folic acid, μg | 86.17 (P < 0.001) | 145.437 (P < 0.001) | 59.267 (P < 0.001) |
| Iron, mg | 6.729 (P < 0.001) | 11.456 (P < 0.001) | 4.727 (P < 0.001) |
| Magnesium, mg | 135.242 (P < 0.001) | 223.009 (P < 0.001) | 87.767 (P < 0.001) |
| MUFA, g | 0.010 (P < 0.001) | 0.019 (P < 0.001) | 0.008 (P < 0.001) |
| Niacin, mg | 10.132 (P < 0.001) | 17.711 (P < 0.001) | 7.579 (P < 0.001) |
| n-3 fatty acid, g | 0.752 (P < 0.001) | 1.301 (P < 0.001) | 0.549 (P < 0.001) |
| n-6 fatty acid, g | 6.273 (P < 0.001) | 10.922 (P < 0.001) | 4.648 (P < 0.001) |
| Protein, g | 0.029 (P < 0.001) | 0.054 (P < 0.001) | 0.025 (P < 0.001) |
| PUFA, g | 0.007 (P < 0.001) | 0.012 (P < 0.001) | 0.005 (P < 0.001) |
| Riboflavin, mg | 0.871 (P < 0.001) | 1.516 (P < 0.001) | 0.644 (P < 0.001) |
| Saturated fat, g | 0.008 (P < 0.001) | 0.015 (P < 0.001) | 0.007 (P < 0.001) |
| Selenium, μg | 39.841 (P < 0.001) | 72.439 (P < 0.001) | 32.598 (P < 0.001) |
| Thiamin, mg | 0.680 (P < 0.001) | 1.164 (P < 0.001) | 0.484 (P < 0.001) |
| Vitamin A, RE | 460.511 (P < 0.001) | 703.562 (P < 0.001) | 243.051 (P < 0.001) |
| Vitamin C, mg | 58.425 (P < 0.001) | 91.183 (P < 0.001) | 32.759 (P < 0.001) |
| Vitamin D, μg | 2.906 (P < 0.001) | 4.760 (P < 0.001) | 1.854 (P < 0.001) |
| Vitamin E, mg | 5.084 (P < 0.001) | 7.474 (P < 0.001) | 2.390 (P < 0.001) |
| Zinc, mg | 4.913 (P < 0.001) | 8.817 (P < 0.001) | 3.904 (P < 0.001) |

Note: All the food parameter are continuous variables, the Newman-Keuls method was conducted as the post-hoc testing, reporting the absolute value of the differences and p-values.
